# Clinical implications of pediatric biliary intraepithelial neoplasia diagnosed from a choledochal cyst specimen

**DOI:** 10.1186/s12957-024-03384-8

**Published:** 2024-04-20

**Authors:** Sujin Gang, Hyunhee Kwon, In Hye Song, Jung-Man Namgoong

**Affiliations:** 1 Department of Pediatric Surgery, Asan Medical Center, 88, Olympic-ro 43-gil, Songpa-gu Seoul, 05505 Republic of Korea; 2 Department of Pathology, Asan Medical Center, 88, Olympic-ro 43-gil, Songpa-gu Seoul, 05505 Republic of Korea

**Keywords:** Biliary intraepithelial neoplasia, Cholangiocarcinoma, Choledochal cysts, Retrospective, Pediatric

## Abstract

**Background:**

Biliary intraepithelial neoplasia (BilIN), a noninvasive precursor of cholangiocarcinoma, can manifest malignant transformation. Since cholangiocarcinoma (CCA) may progress due to chronic inflammation in the bile ducts and gallbladder, choledochal cysts are considered a precursor to CCA. However, BilIN has rarely been reported in children, to date.

**Methods:**

We reviewed medical records of patients (< 18 years of age, *n* = 329) who underwent choledochal cyst excision at Asan Medical Center from 2008 to 2022. BilIN was diagnosed in 15 patients. Subsequent analyses were performed of the demographics, surgical procedures, clinical course, and outcomes in these patients. Subgroup analysis and multivariate logistic regression test were performed to identify factors influencing BilIN occurrence.

**Results:**

The mean age of the patients included in our study was 40.1 ± 47.6 months. In 15 patients, BilIN of various grades was diagnosed. Todani type I was prevalent in 80% of the patients. The median age at surgery was 17 months. During a mean follow-up of 63.3 ± 94.0 months, no adverse events such as stone formation in the remnant intrapancreatic common bile duct and intrahepatic duct or cholangiocarcinoma were observed, indicating a favorable outcome until now.

**Conclusions:**

The potential progression of choledochal cysts to BilIN in children was demonstrated. These results could underscore the importance of early and comprehensive excision of choledochal cysts, including resection margins for associated lesions and more thorough postoperative surveillance in patients with or at risk of BilIN.

## Background

Choledochal cyst (CC) is defined by the abnormal biliary tract dilation, primarily occurring in the common bile duct. It is more common in Asian populations, with an incidence of approximately one in 1,000 individuals [1]. The most commonly used classification system is the Todani classification, which is based on morphology and includes five types [2, 3]. Type I is the most common (75–85%), followed by type IV (13%), type III (4%), type II (3%), and type V (1%).

The mechanisms underlying the formation of CC remain unknown. However, a combination of factors is thought to be involved [1]. Congenital weakness of the bile duct wall is a predisposing factor, indicating a genetic component underlying this condition. Additionally, bile duct obstruction may play a role in triggering the formation of CCs [4]. Reflux of pancreas enzyme through anomalous pancreaticobiliary ductal union (APBDU) is known to be a contributing factor. When the bile duct is obstructed or manifests abnormal pressure, structural changes can occur, potentially contributing to cyst formation. Chronic inflammation may also weaken the bile duct walls, further promoting the development of cystic changes within the duct.

In this context, CCs are associated with malignancies of the biliary system [5]. The development of biliary tract carcinoma is associated with chronic inflammation, contributing to carcinogenesis in a stepwise manner. Biliary system carcinomas follow a sequential carcinogenic pathway, beginning with metaplasia, progressing to biliary intraepithelial neoplasia (BilIN), and culminating in carcinoma. BilIN has been found in a significant percentage of bile duct carcinoma cases, ranging between 10 and 45% [6].

BilIN is considered a non-invasive precursor lesion of cholangiocarcinoma (CCA) and has the potential to transform into an invasive cancer [7, 8]. BilINs typically manifest as predominantly flat, micropapillary, or pseudopapillary in situ lesions, progressing to CCA through a multistep carcinogenic process [8]. The pathogenesis of BilIN in patients with CC is well-established in adults. Sporadic reports of BilIN in the pediatric population have also been published. However, the clinical significance of BilIN in children has not yet been thoroughly investigated or elucidated.

In 2022, we identified BilIN in the pathology report of an CC specimen from a 7-year-old girl. This finding prompted us to conduct a comprehensive review of the pathological results of patients who underwent surgery at our hospital. BilIN was confirmed in samples from 17 patients between January 2000 and June 2023. This finding highlighted the need for further investigation into the clinical implications of BilIN in pediatric patients with CC.

In 2006, Tanaka et al. reported the case of an 11-year-old male diagnosed with CCA after a CC. Their study was included in a 2018 literature review by Newsome et al. [9, 10]. This indicates the need for considering the clinical significance of BilIN in younger patients. Therefore, in this study, we investigated the clinical characteristics, progression, and potential risk factors associated with BilIN identified in CC specimens. We aimed to gain insights into the clinical implications of this condition in pediatric patients.

## Methods

This study was retrospectively conducted including pediatric patients (patients aged less than 18 years) who underwent CC excision and Roux-en-Y hepaticojejunostomy procedures at the Department of Pediatric Surgery in Asan Medical Center between 2000 and 2022. BilIN was detected in the surgically excised CCs from15 out of 329 patients (4.5%). The mean age at the time of surgery of all patients included in this study was 40.1 ± 47.6 months (Range: 0–286 months), with 225 (77.5%) being female patients. We identified two more patients who underwent surgery in 2023. However, we excluded them due to a lack of follow-up information.

We collected comprehensive data, including demographic information, surgical procedures, and subsequent follow-ups from patients’ medical charts. The follow-up period was determined based on the most recent outpatient visit date. Complications were assessed using the Clavien–Dindo classification system.

This study was conducted in accordance with the principles of the Helsinki Declaration. It was approved by the Institutional Review Board of Asan Medical Center (approval No: 2023 − 0837). Informed consent was waived owing to the retrospective nature of the study.

### Surgical procedure

Surgery was performed using either an open or minimally invasive approach, depending on the preference of the surgeon and the timing of the operation. The open approach involved a right subcostal incision, whereas the minimally invasive approach used a four-port system. The CC was identified through abdominal exploration, and both the cystic duct and artery were identified and ligated before cyst dissection, using them as reference points for dissection.

In some cases, for visual confirmation of the hepatic artery during dissection, the cyst was cut horizontally to avoid damage to the artery. Proximal resection was performed at the level of the normal common bile duct to ensure complete removal of the cyst. Distally, the bile duct was dissected toward the inside of the pancreatic head; after confirming the normal duct, it was cut and ligated using a Hem-o-lock® clip (Teleflex, Wayne, PA, USA).

An end-to-side jejunojejunal (JJ) anastomosis was performed approximately 40 cm distal to the Treitz ligament, preserving a 40 cm length of jejunum between the JJ and the HJ anastomoses. The HJ anastomosis was retrocolic. After confirming the absence of any leaks at the HJ anastomosis site, a drain is placed behind the anastomosis; the operation was terminated with skin closure.

### Histopathological examination

The specimens, including the gallbladder and CC, were histopathologically examined. After formalin fixation, the gallbladder and CCs were opened and examined for grossly visible nodules or masses on their luminal surfaces. Representative sections were obtained from the specimens along the longitudinal axis, and paraffin blocks were prepared. The number of submitted blocks in each case was determined based on the size of the CC. The median number of submitted blocks was 2 (range: 1—5) for CC with one additional block for gallbladder. The initial histopathological diagnoses were reported by general gastrointestinal or hepatobiliary pathologists. In this study, a pathologist with 7 years of experience in gastrointestinal and hepatobiliary pathology reviewed the histological slides to confirm the presence of BilIN and graded the specimens according to the current grading system [11].

### Statistical analysis

All statistical analyses were performed using SPSS (version 26), and multivariate regression analysis were conducted to identify the risk factors associated with BilIN. Statistical significance was set at *p* < 0.05.

## Results

Table 1 lists the demographic characteristics of the patients upon admission. Most patients were female, constituting 66.7% of the total, and five patients (33.3%) had undergone surgical procedures during the neonatal period. The most common cause of admission was abdominal pain, observed in eight patients (53.3%). Diarrhea or vomiting occurred in two patients, respectively, owing to concurrent pancreatitis.

**Table 1:** Initial characteristics of the study participants (*n* = 15)

	Number of patients	%
**Sex**		
Male	5	33.3
Female	10	66.7
**Neonates**	5	33.3
**Underlying disease**	3	20.0
TOF (Tetralogy of Fallot)	1	6.7
Hereditary Pancreatitis	1	6.7
Aplastic anemia (s/p allo-PBSCT)	1	6.7
**Initial symptoms**		
Abdominal pain	8 (Vomiting, 3)	53.3
Incidental findings	2	13.3
Fetal ultrasound	4	26.6
Diarrhea	1	6.7
Vomiting	1	6.7

PBSCT, peripheral blood stem cell transplantation

The diagnosis was primarily established through ultrasonography (US) and magnetic resonance cholangiopancreatography (MRCP). Todani type I CC was the most prevalent, accounting for 80.8% of patients (*n* = 12; Table 2), and 11 (73%) patients were identified as having an APBDU. However, the presence of APBDU remained unclear in 4 patients. Four patients underwent preoperative obstructive symptoms necessitating endoscopic retrograde cholangiopancreatography (ERCP). Abnormalities in liver function tests were rare. Pancreatitis was commonly observed.

**Table 2:** Preoperative findings of the study participants

	Number of patients	%
**Diagnosis**		
US (Ultrasonography)	12	80.0
CT (Computed tomography)	7	46.7
MRCP	12	80.0
**Imaging findings**		
Type		
I	12	80.0
IVa	2	13.3
Cyst (largest diameter, cm)	3.3 ± 1.3	
APBDU		
Yes	11	73.3
Unknown	4	26.7
Perforation/Panperitonitis	1/1	6.7
**Preoperative ERCP**	4 (ENBD insertion, *n* = 2)	26.7
**Preoperative laboratory findings**		
AST (*n* = 14)	38.5 [33.0;58.0]	IU/L
ALT (*n* = 14)	26.5 [11.0;205.0]	IU/L
B.bilirubin (*n* = 14)/D.biliirubin (*n* = 9)	2.5 [0.7;7.3]/0.5 [0.4;1.0]	mg/dL
Amylase/Lipase (*n* = 11)	118.0 [62.0;146.0]/128.0 [60.5;441.0]	U/L
Leukocytosis (*n* = 14)	4 (28.6%)	
CRP (*n* = 14)	40.1 [0.1;0.4]	mg/dL
SIRS (*n* = 14)	0	

MRCP, magnetic resonance cholangiopancreatography; APBDU, anomalous pancreaticobiliary ductal union; ERCP, endoscopic retrograde cholangiopancreatography; ENBD, endoscopic nasobiliary drainage; AST, aspartate transferase; ALT, alanine transaminase; B. bilirubin; D.bilirubin, direct bilirubin; CRP, C-reactive protein; SIRS, systemic inflammatory response syndrome

Surgery was performed at approximately 17 months of age. A minimally invasive approach was performed in most patients (Table 3). Necrotic changes with perforation of CCs and cirrhotic liver changes were observed in three patients each, necessitating follow-up. Pancreatitis detected during operation was the most frequently observed concurrent condition in 47% of the patients.

**Table 3:** Operation-related data

	Number of patients	%
**Age at operation (months)**	17.0 [6.0;47.0]	
**BMI at operation (kg/m** ^**2**^ **)**	14.4 ± 2.0	
**Approach**		
Open	1	6.7
Laparoscopy	8	53.3
Robotic	3	20.0
Conversion	1	6.7
**Operation time (minutes)**	281.6 ± 44.0	
**Transfusion**	0	
**OP findings**		
Perforation	2	13.3
Necrosis	1	6.7
Liver cirrhosis	3	20.0
**Pancreatitis**	**7**	**46.7**
Pericystic inflammation	3	20.0
Anastomosis diameter (mm)	9.1 ± 5.5	(*n* = 14)

BMI, body mass index; OP, operative

Among the 15 patients diagnosed with BilIN, multifocal lesions were identified in nine (60.0%), whereas margin involvement was not observed (Table 4). In three patients, margin involvement was obscure due to specimen fragmentation. Notably, one patient (patient 11 in Table 4), who aged less than 1 year, required remnant cyst excision owing to cholangitis following an incomplete initial excision, resulting in the discovery of high-grade BilIN. This case underscores the possibility of BilIN progression, even in children under 1 year of age, when severe chronic inflammation persists. Patients with high-grade BilIN had relatively larger lesions than did those with low-grade BilIN. This implicates that the progression of BilIN resulted from chronic inflammation.

**Table 4:** Pathologic results (*n* = 15)

Patient	Age at OP (month)	APBDU	Gallbladder	Choledochal cyst	Maximum size (cm)	Multifocality	Margin involvement
1	14.6	Unknown		BilIN, HG	0.7	N	N
2	41	Yes		BilIN, Gr2	0.3	N	N
3	0	Unknown		BilIN, Gr2	0.1	N	N
4	42	Yes	BilIN, Gr1		0.1	N	N
5	44	Yes		BilIN, Gr1	0.1	Y	N
6	12	Yes	BilIN, Gr2		0.2	N	N
7	55	Yes	BilIN, Gr2	BilIN, Gr2	0.1	Y	N
8	13	Yes		BilIN, HG	0.1	Y	N
9	17	Yes		BilIN, HG	0.5	N	N
10	50	Yes		BilIN, Gr2	0.1	Y	N
11	0	Yes		BilIN, HG	0.6	Y	U
12	85	Yes		BilIN, HG	0.2	Y	N
13	0	Unknown	BilIN, HG	BilIN, HG	0.7	Y	U
14	0	Unknown	BilIN Gr1		0.1	Y	N
15	94	Yes		BilIN, Gr1	0.2	Y	U

OP, operation; APBDU, anomalous pancreaticobiliary ductal union; BilIN, biliary intraepithelial neoplasia; Gr, grade; HG, high grade; U, uncheckable due to fragmented specimen; Y, yes; N, no

Notably, all patients are undergoing outpatient follow-up recently, and as of this writing, they have not experienced postoperative complications of Clavien–Dindo grade ≥ 3 (Table 5). US assessments were used for follow-up observation, revealing minimal intrahepatic duct dilatation in two patients and fatty liver in one patient. No other chronic complications, such as HJ structures and intrahepatic duct (IHD) stone, were reported.

**Table 5:** Clinical outcomes

Patient	Postoperative complication	F/U (months)	F/U imaging	Results	AST (IU/L)	ALT (IU/L)	ALP (IU/L)	T.Bil (mg/dL)
**1**		151	US	normal				
**2**	Late pancreatitis (Gr2)	71	US	normal				
**3**		80	US	Prominent periportal echogenicity in the liver	35	23	227	0.2
**4**		93	US	pneumobilia	30	14	390	0.5
**5**	Late pancreatitis (Gr1)	72	US	Diffuse increased liver echogenicity suggesting fatty liver	25	28	300	0.5
**6**	Vomiting (Gr1)	51	US	Stable minimal proximal right IHD dilatation	53	24	243	0.4
**7**		56	US	Small pneumobilia	33	16	244	0.3
**8**		53	US	normal	37	9	192	0.4
**9**		31	US	normal	26	13	136	0.4
**10**		26	US	mild prominent proximal IHD	26	9	186	0.2
**11**		12	US	normal	51	35	281	0.2
**12**		3	US	normal	29	5	240	0.1
**13**		151	US	normal				
**14**		71	US	normal				
**15**	Fluid collection (Gr 1)	80	US	Prominent periportal echogenicity in the liver	35	23	227	0.2

F/U, follow-up; AST, aspartate transferase; ALT, alanine transaminase; ALP, alkaline phosphatase; T.Bil, total bilirubin; Gr, grade; US, ultrasonography; IHD, intrahepatic duct

A subsequent subgroup analysis was performed to investigate the factors influencing the development of BilIN (Table 6). The presence of APBDU in patients with BilIN did not differ from those without it (*p* = 0.680). Only serum lipase elevation was observed in patients with BilIN (*p* = 0.034). This finding indicates that chronic inflammation may contribute to histopathological alterations such as BilIN. We additionally performed multivariate logistic regression analysis. None of the factors included in Table 6 were found, statistically, to contribute to BilIN occurrence.

**Table 6:** Sub-group analysis of clinical characteristics

That	Normal pathology	BilIN	p-value
	(*n* = 314)	(*n* = 15)	
**Sex**			0.342
Male	69 (22.0%)	5 (33.3%)	
Female	245 (78.0%)	10 (66.7%)	
**Age at operation (months)**	24.0 (4.2–54.0)	17.0 (6.0–47.0)	0.708
**APBDU**			0.680
No	38 (12.3%)	1 (6.7%)	
Yes	182 (58.7%)	11 (73.3%)	
Unknown	90 (29.0%)	3 (20.0%)	
**Preoperative lab findings**			
AST (IU/L)	34.0 [28.0;50.0]	38.0 [31.5;46.0]	0.465
ALT (IU/L)	20.0 [12.0;54.0]	17.0 [10.5;33.0]	0.550
T.bil (mg/dL)	0.6 [ 0.4; 2.5]	0.8 [ 0.5; 4.8]	0.285
D.bil (mg/dL)	0.3 [ 0.1; 0.8]	0.5 [ 0.3; 1.0]	0.149
Amylase (U/L)	66.0 [32.0;112.0]	121.5 [45.0;154.0]	0.108
Lipase (U/L)	36.5 [20.0;90.5]	110.0 [47.0;693.0]	**0.034**
Leukocytosis			0.580
No	229 (73.4%)	10 (66.7%)	
Yes	83 (26.6%)	5 (33.3%)	

BilIN, biliary intraepithelial neoplasia; CC: choledochal cyst; APBDU, anomalous pancreaticobiliary ductal union; AST, aspartate transferase; ALT, alanine transaminase; T.bil, total bilirubin; D.bil, direct bilirubin. Bold indicates a significant p-value (*p* < 0.05)

## Discussion

BilIN is diagnosed based on histological observation, indicating a microscopic precancerous stage in the biliary tract, corresponding to carcinoma in situ. This is accepted as a precursor lesion of CCA, particularly large duct type, which is known to have a poor prognosis [12]. Early precursors of CCA also encompass intraductal papillary neoplasms of the bile ducts, biliary mucinous cystic neoplasms, bile duct adenoma, and von Meyenburg complexes [13].

Owing to the lack of distinct mass formation, BilIN cannot be definitively identified on diagnostic imaging. It is incidentally encountered proximal to invasive carcinoma in most cases, the prevalence of isolated BilIN, unaccompanied by invasive carcinoma, remains uncertain.

These lesions may develop owing to chronic inflammation of the bile ducts. Numerous biliary epithelial cell-associated molecular pathways have been identified. For example, Kirsten rat sarcoma 2 viral oncogene homolog gene expression has been observed in approximately 40% of BilIN cases, and tumor protein 53 mutations have also been reported, particularly in advanced stages [14]. Factors such as cholestasis within the cyst and pancreatic enzyme reflux into the bile duct through APBDU can lead to the production of cytotoxic substances such as lysolecithin, which may contribute to malignant transformation in chronic inflammation of the bile duct [15, 16].

Histologically, BilIN is confirmed in the biliary epithelium adjacent to the invasive adenocarcinoma. Grossly, BilIN often manifests with unclear features such as mucosal thickening, granularity, and subtle alterations in mucosal color. However, as the BilIN grade increases, changes within epithelial cells become more pronounced [13]. In 2005, the grading of BilIN was proposed as a three-grade classification: BilIN-1, BilIN-2 and BilIN-3. However, in 2015, the classification was revised to a two-tier system: low-grade BilIN and high-grade BilIN, aiming to achieve higher concordance rates and practicality [11, 17]. The current WHO tumor classification also recommends a two-tier system [18].

**Fig. 1:**
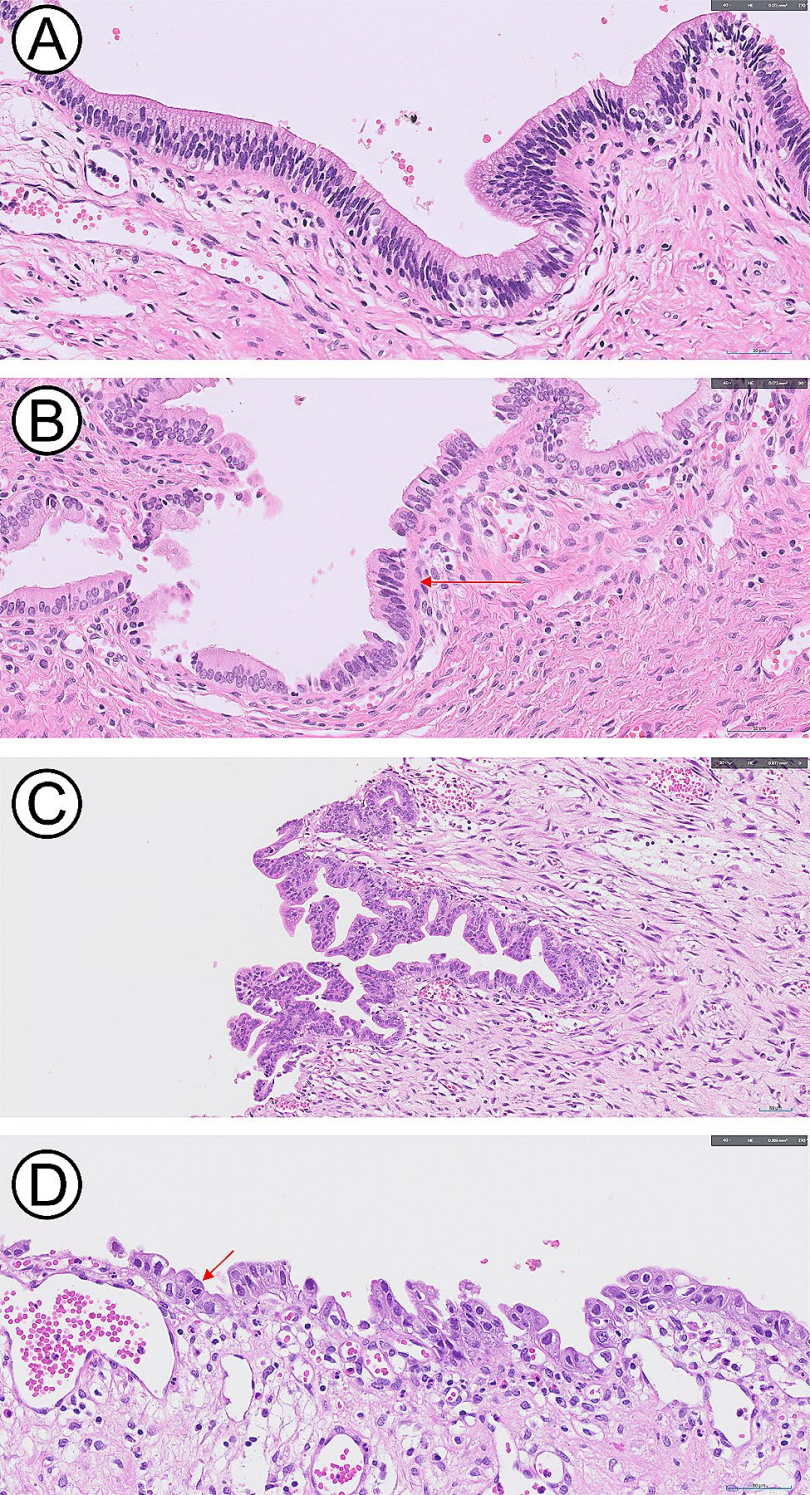
Representative microscopic images of low- and high-grade biliary intraepithelial neoplasia (BilIN) arising from a choledochal cyst. (**A** & **B**) Low-grade BilIN showing a flat growth pattern, nuclear pseudostratification (**B**, arrow), mild nuclear enlargement, increased nuclear-to-cytoplasmic ratio, and hyperchromasia (**A** & **B**, hemotoxylin and eosin [H&E], ×400). (**C** & **D**) High-grade BilIN shows a more complex micropapillary pattern, marked nuclear atypia, and frequent mitosis (**D**, arrow) (**C**, H&E, ×200; **D**, H&E, ×400)

Low-grade BilIN (classified as BilIN grades I and II) exhibits mild cytological atypia characterized by hyperchromatic nuclei, prominent nucleoli, a slight increase in the nucleus-to-cytoplasm ratio, and minor nuclear pseudostratification, while retaining nuclear polarity. In contrast, high-grade BilIN (BilIN grade III) displays severe nuclear atypia, irregular and pleomorphic nuclei, several mitotic figures, and complete loss of nuclear polarization with complex stratification. Additionally, high-grade BilIN may exhibit features such as pseudopapillary eosinophilic epithelium or micropapillary structures [12]. The pathological findings in our patients are consistent with them (Fig. 1).

**Fig. 2:**
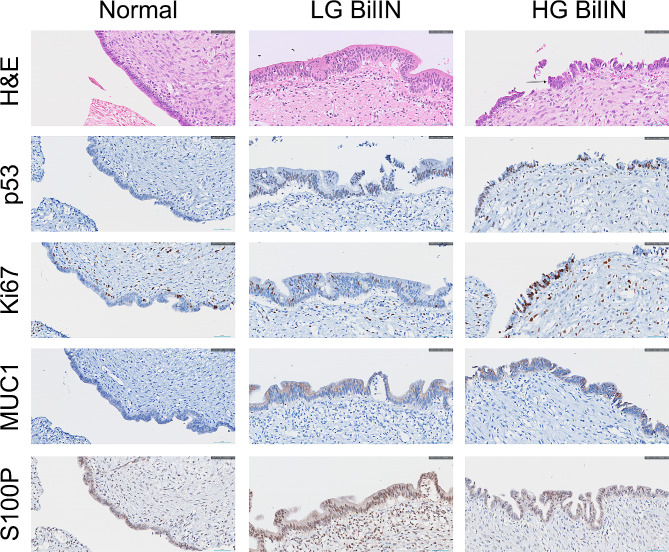
Hematoxylin and eosin (H&E) and immunohistochemical staining. H&E staining and immunohistochemical staining for p53, Ki67, MUC1, and S100P in normal bile duct tissue and low- and high-grade biliary intraepithelial neoplasia (BilIN) arising from a choledochal cyst. BilINs exhibit increased expressions of p53, Ki67, and MUC1 compared to those in normal biliary epithelial tissue. High-grade BilIN displays higher levels of p53 and Ki67 expression than low-grade BilIN. S100P expression does not differ between cases.

Immunohistochemistry can be applied to differentiate between low- and high-grade BilINs. Molecules such as p21, p53, cyclin D1, S100P, MUC1, and MUC5AC are increased in patients with BilIN [15]. However, distinguishing preinvasive lesions from invasive ones is challenging. Currently, three markers, S100, p53, and p16 are used for this purpose [12]. In this study, increased expression of p53, Ki67, and MUC1 were observed in all BilIN specimens (Fig. 2). In cases of high-grade BilIN, expression of p53 and Ki67 was relatively more prominent. This indicates more active carcinogenesis. However, no significant differences in S100 expression were observed. This finding differs from that in previous reports describing increased S100 expression in BilIN, which increases as BilIN grade increases [15]. In addition, distinguishing preinvasive lesions from invasive carcinomas remains difficult. Zen et al. comfirmed the interobserver agreement in distinguishing between BilIN and reactive lesions, demonstrating a moderate diagnostic sensitivity of this method [16].

In 1995, in a study that established CC as a risk factor for CCA, Stain et al. reported that tumor identified in 26% of patients with CC [19]. However, the histology of these tumors was diverse, with adenocarcinoma being the most common, followed by adenosquamous carcinoma, squamous cell carcinoma, and rhabdomyosarcoma. The incidence of these tumors increased with patient age.

In patients diagnosed with BilIN, no occurrence of stones in the intrahepatic duct or intrapancreatic head portion bile duct, nor progression to cancer, has been observed in the postoperative course up to the present. However, this is suggested to be due to the relatively short follow-up period. Performing subgroup analysis of data of 329 patients who underwent surgery between 2008 and 2022, factors such as age at the time of surgery, preexisting cholangitis or pancreatitis, and presence of APBDU were considered (Table 6). Serum liver enzyme level was not significantly different between the two groups. Of note, the serum amylase level was higher in patients with BilIN, without showing statistical significance (*p* = 0.108). However, serum lipase was significantly higher in the patients with BilIN (*p* = 0.034), suggesting that it could reflect the results of recurrent chronic pancreatitis. We additionally performed multivariate logistic regression analysis. However, none of factors listed in Table 6 and type of CC by Todani’s classification were found to significantly influence BilIN occurrence.

In 2023, Gao et al. developed a nomogram to predict premalignant lesions in patients with CC (cholangiocarcinoma); they reported BilIN in pediatric population for the first time in this publication [20]. In this study involving 210 patients with CC, age at operation, recurrent episodes of biliary pancreatitis, duration of symptoms, cyst diameter, and history of biliary operations before excision surgery were found to be statistically significant risk factors. Age at operation was not different between patients with BilIN and those without it in our study. However, the increase in serum amylase level indirectly could suggest a relationship between recurrent biliary pancreatitis and stepwise malignant transformation of the extrahepatic bile duct.

In a retrospective study conducted by Katabi et al., BilIN was identified in 28.5% of patients (age range, 11–67 years) who underwent surgery for CCs at the Memorial Sloan Kettering Cancer Center [6]. We noted the presence of BilIN in a CC specimen obtained from a 7-year-old girl in 2022. Except her, the youngest patient reported with a single case of BilIN was a 21-year-old female in whom high-grade BilIN was confirmed as a Type IA CC. Based on the 2006 report by Tanaka et al., the youngest patient diagnosed with CCA within a CC was 11, and considering the age of our aforementioned 7-year-old patient, an awareness of the clinical significance of BilIN in younger patients is necessary [10].

As of now, there have been no reports of progression of residual bile duct cancer to CCA in any of the patients enrolled. However, since the oldest patient was 17, long-term follow-up seems necessary. Considering the nature of BilIN, which is not visible in imaging, further research is necessary to develop tests performed during follow-up for early detection of carcinogenesis. In addition, well-designed prospective studies, including patient population with complete excision of CC and histopathological diagnosis of BiIIN, are needed to investigate this disease further. It is related with limitation of this study which was a retrospective, single-center study based on incidental diagnostic results.

The clinical significance and treatment standards of BilIN have not been fully established, even in the adult population. Therefore, conducting a study to determine the clinical significance of the lesion through a large-scale analysis, including a review of histopathological examinations, may be helpful. This is particularly meaningful considering the relatively short history of successful CC excisions in children and the increased risk of carcinogenesis in older patients.

Currently, little is known about the clinical implications of BilIN other than that it is a precancerous lesion [7, 8, 21]. Considering the possibility of tumorigenesis in residual BilIN, complete resection of high-grade BilIN is indicated during CCA excision in the adult population. Therefore, when high-grade BilIN is confirmed in a pediatric CC specimen, surgeons must assess margin involvement and consider the necessity of additional resection. As no established consensus on treatment guidelines for BilIN exists, developing a strategy for detecting future carcinogenesis is also crucial [22].

To better understand the clinical implications of BilIN in pediatric patients, future large-scale prospective studies are essential to identify risk factors and establish long-term follow-up guidelines. This will help verify the clinical significance of BilIN and guide its management in children.

Our findings indicated that the possibility of BilIN should not be overlooked in children, particularly in those with risk factors such as preoperative chronic pancreatitis. Surveillance of CCA should not be omitted in this population. Furthermore, our findings promote the need for early and complete resection of CCs, potentially important for preventing neoplastic changes in the biliary system. Furthermore, in patients at high risk for BilIN, verifying the presence of BilIN at the resection margins is essential to ensure complete resection.

## Data Availability

No datasets were generated or analysed during the current study.

## Abbreviations

APBDU: Anomalous Pancreaticobiliary Ductal Union
BilIN: Biliary Intraepithelial Neoplasia
CCA: Cholangiocarcinoma
CC: Choledochal Cyst
HJ: Hepaticojejunal
JJ: Jejunojejunal
MRCP: Magnetic Resonance Cholangiopancreatography
SPSS: Statistical Package for the Social Sciences
US: Ultrasonography
